# The regulatory role of adipocyte mitochondrial homeostasis in metabolism-related diseases

**DOI:** 10.3389/fphys.2023.1261204

**Published:** 2023-10-18

**Authors:** Hongbing Song, Xiaohan Zhang, Jing Wang, Yanling Wu, Taimin Xiong, Jieqiong Shen, Ruiyi Lin, Tianfang Xiao, Weimin Lin

**Affiliations:** College of Animal Sciences (College of Bee Science), Fujian Agriculture and Forestry University, Fuzhou, Fujian, China

**Keywords:** adipocyte, adipose tissue, mitochondria, obesity, metabolic syndrome

## Abstract

Adipose tissue is the most important energy storage organ in the body, maintaining its normal energy metabolism function and playing a vital role in keeping the energy balance of the body to avoid the harm caused by obesity and a series of related diseases resulting from abnormal energy metabolism. The dysfunction of adipose tissue is closely related to the occurrence of diseases related to obesity metabolism. Among various organelles, mitochondria are the main site of energy metabolism, and mitochondria maintain their quality through autophagy, biogenesis, transfer, and dynamics, which play an important role in maintaining metabolic homeostasis of adipocytes. On the other hand, mitochondria have mitochondrial genomes which are vulnerable to damage due to the lack of protective structures and their proximity to sites of reactive oxygen species generation, thus affecting mitochondrial function. Notably, mitochondria are closely related to other organelles in adipocytes, such as lipid droplets and the endoplasmic reticulum, which enhances the function of mitochondria and other organelles and regulates energy metabolism processes, thus reducing the occurrence of obesity-related diseases. This article introduces the structure and quality control of mitochondria in adipocytes and their interactions with other organelles in adipocytes, aiming to provide a new perspective on the regulation of mitochondrial homeostasis in adipocytes on the occurrence of obesity-related diseases, and to provide theoretical reference for further revealing the molecular mechanism of mitochondrial homeostasis in adipocytes on the occurrence of obesity-related diseases.

## 1 Introduction

Adipose tissue plays a key role in maintaining whole-body energy metabolic homeostasis. Excessive energy intake is converted into triglyceride and stored in white adipose tissue. Later, the triglycerides are converted into free fatty acids, which are utilized by other organs through circulation when animals experience a lack of nutritional intake (Bartelt and Heeren, 2014). Moreover, adipose tissue is one of the most important endocrine organs, secreting over 700 adipokines, such as adiponectin (Liu et al., 2016) and leptin (Harris, 2014). These hormones regulate the growth, development, and metabolism of other tissues and organs (Sun et al., 2011). In addition, dysfunction of adipose tissue is associated with metabolic diseases, including diabetes, insulin resistance, and obesity (Carobbio et al., 2017).

The mitochondria are the main location for energy metabolism in eukaryotic cells. In recent years, there has been growing interest in the relationship between adipocytes and their mitochondria. Studies have shown that mitochondria have great effects on adipose tissue resident cells, including adipocytes and adipocyte progenitors (Zhu et al., 2022). Numerous studies have shown that normal mitochondrial function is a prerequisite for adipose tissue to function as an energy storage location and endocrine organ (De Pauw et al., 2009; Kusminski and Scherer, 2012; Vernochet et al., 2014). However, dysfunction in adipocytes, such as a decrease in the synthesis and secretion of adiponectin, can be induced by dysregulated mitochondrial function (Koh et al., 2007; Wang C. H. et al., 2013; Koh et al., 2015).

Therefore, it is significant to understand the relationship between the biological characteristics of mitochondrial and adipocytes. In this paper, we concentrate on the structure of adipocyte mitochondrial, their association with other organelles, quality control mechanisms, and metabolism to shed to light on their impact on adipose tissue and related diseases.

## 2 Adipocyte mitochondrial structure

### 2.1 Adipocyte mitochondrial physical structure

Mitochondria are surrounded by two layers of lipid bilayer, consisting of the outer mitochondrial membrane (OMM) and the inner mitochondrial membrane (IMM). There are differences in their composition. The OMM has a lipid composition similar to that of the eukaryotic cell membrane. However, the protein-to-lipid ratio of the IMM is higher than that of the OsMM (Ernster and Schatz, 1981). The IMM is concave and forms high-density folds in the matrix, known as cristae, which expand the surface area and are beneficial for generating ATP (Ernster and Schatz, 1981). Varghese et al. reported that in the cardiac intramuscular fat of mice under normal conditions, cristae of mitochondria attached to lipid droplets were perpendicular to the tangent of the contact surface (Varghese et al., 2019). They also found that the number of vertical cristae decreased after fasting. Interestingly, intraperitoneal injection of CL316,243, an activator of adrenaline receptor, increased the number of vertical cristae (Varghese et al., 2019). However, the molecular mechanism responsible for the change in direction of droplet-mitochondrial cristae remains unclear. It is worth mentioning that mitochondrial shaping proteins play a crucial role in maintaining the morphology and function of cristae. These proteins include optic atrophy 1 (OPA1), myeloid cell leukemia 1 (MCL1), prohibitin 1 (PHB1), stomatin-like protein 2 (SLP2), and ATP synthase (ATPase) (Cogliati et al., 2016; Ikon and Ryan, 2017). OPA1 is among them and is associated with the browning of white adipocytes (Bean et al., 2021). Overexpression of OPA1 is beneficial for the expansion and browning of white adipose tissue, while the adipocyte-specific deletion of OPA1 limits the beige differentiation of preadipocytes (Bean et al., 2021).

Phospholipids are the main component of membranes. Phospholipid synthesis and remodeling are necessary for the formation, enlargement, maintenance, and function of the plasma membranes and organelles, such as lipid droplets, endoplasm reticulum, and mitochondria (van Meer et al., 2008). In eukaryotic cells, mitochondria can synthesize a portion of the phospholipids required for their own structure and function. However, most of the phospholipids are synthesized in the endoplasmic reticulum and transported into the mitochondria via mitochondria-related endoplasmic reticulum (Fagone and Jackowski, 2009). Cardiolipin is one of the most vital phospholipids. Cardiolipin not only functions as a component of membranes, but also plays a vital role in mitochondria-regulated apoptosis and other cellular processes (Mejia and Hatch, 2016). Research suggests that phospholipids play a vital role in the physiological processes of adipocytes, particularly cardiolipin. Cardiolipin may have a specific function in brown and beige adipocytes (Sustarsic et al., 2018). Cardiolipin interacts with creatine kinase, which controls the thermogenic futile cycle in beige adipocytes (Chouchani et al., 2016). Cardiolipin tightly binds with uncoupling protein 1 (UCP1), which is beneficial for the correct folding of cardiolipin (Hoang et al., 2013). The prediction of binding sites of cardiolipin on UCP1 is closely associated with the cysteine residue, and thermogenesis ability is promoted by sulfonylation of this free radical (Chouchani et al., 2016).

### 2.2 Adipocyte mitochondrial DNA

In humans, mitochondrial DNA (mtDNA) encodes 37 genes, which consist of 22 tRNA-encoding genes, 2 rRNA-encoding genes, and 13 genes that encode proteins involved in the electron transfer chain (Taanman, 1999). Due to the absence of protective structures such as histones and introns, and its proximity to ROS-producing site, mtDNA is highly susceptible to oxidative damage (Sampath et al., 2011). In mammals, damaged mtDNA is primarily repaired through the base excision repair pathway. This pathway involves key roles played by DNA glycosylases such as Nei like DNA glycosylase 1 and 2 (NEIL1 and NEIL2); Oxoguanine DNA glycosylase 1 (OGG1); *N*th like DNA glycosylase 1 (NTH1) (Dizdaroglu, 2005). Mice with a deletion of NEIL1 (NEIL−/−) or heterozygous mice (NEIL^+/−^) exhibited severe obesity, dyslipidemia, and fatty liver disease (Vartanian et al., 2006). Consistently, mice fed a high-fat diet and with a deletion of NEIL1 gained more body fat and weight, and exhibited fatty liver degeneration compared to wild-type mice (NEIL^+/+^) (Sampath et al., 2011). Interestingly, targeted transgenesis of *OGG1* towards the mitochondrion protected mice against obesity induced by a high-fat diet. This intervention also improved insulin resistance and reduced adipose tissue inflammation, which was associated with an increase in mitochondrial respiratory capability in adipose tissue (Komakula et al., 2018). The deletion of *OGG1* in mice resulted in the downregulation of genes related to fatty oxidation and the tricarboxylic acid cycle. As a consequence, the mice showed a tendency towards obesity and insulin resistance (Sampath et al., 2012). These results indicate that impaired mitochondrial repair is one of the reasons that contribute to obesity-related diseases, including obesity, insulin resistance, and fatty liver degeneration.

In recent years, there has been increasing attention given to the epigenetics of mtDNA, especially mtDNA methylation. In humans, the level of mtDNA methylation was higher in overweight females compared to lean females (Bordoni et al., 2019). Moreover, the level of mtDNA methylation was found to increase in retinal endothelial cells that were exposed to a high glucose environment. This increase in methylation resulted in decreased transcription levels of mtDNA (Mishra and Kowluru, 2015). However, Corsi considered that a special form of mtDNA methylation found in platelets of overweight or obese patients could predict the occurrence of cardiovascular diseases (Corsi et al., 2020). Interestingly, it has been reported that methylation of the mtDNA-encoded NADH dehydrogenase is correlated with the degree of hepatic diseases (Pirola et al., 2013). The data shows that methylation of mtDNA inhibits the expression of genes encoded by mtDNA, which influences the homeostasis of mitochondrial metabolism and can lead to diseases caused by organizational dysfunction. Notably, Wahl confirmed that mtDNA methylation is a consequence of obesity rather than the cause of obesity (Wahl et al., 2017).

In adipocytes, the copy number of mtDNA is regulated by adipogenic genes (reviewed later). However, mtDNA copy number influences cardiovascular disease by regulating methylation of nuclear DNA (nucDNA) (Castellani et al., 2020). Besides, more and more researches indicate that nuclear transcription factors exists in the mitochondria of mammals and may be involved in regulating expression of mtDNA. However, there is still a need to explore additional modes of interaction and the impact of interaction regulation between mtDNA and nucDNA on adipocytes (Leigh-Brown et al., 2010; Szczepanek et al., 2012).

## 3 Connection between mitochondria and other organelle in adipocyte

### 3.1 Peridroplet mitochondria

Lipid droplets are the primary storage location for lipids, surrounded by phospholipid monolayers embedded with internal and peripheral proteins. Emerging research has shown that part of mitochondria is attached to lipid droplets in adipocytes, and have different physiological characteristics from cytoplasmic mitochondria (CM). Proteins located at the linkage site between mitochondria and lipid droplets, such as perilipin 5 (PLIN5), have been observed using a super-resolution microscope to be located at the contact site between lipid droplets and mitochondria (Gemmink et al., 2018). Overexpression of *PLIN5* has been found to reduce lipolysis and β-oxidation, and enforce palmitate binding to triglycerides (Wang et al., 2011). Furthermore, it has been identified that overexpression of *PLIN5* facilitates the recruitment of mitochondria from the cytoplasm to lipid droplets and promotes the expansion of lipid droplets in the ovary, liver, and heart tissue of Chinese hamsters (Wang H. et al., 2013). Additionally, endoplasmic reticulum enzyme DGAT2, which is located in the endoplasmic reticulum and lipid droplet membrane, which has the ability to recruit CM to lipid droplets (Stone et al., 2009). This enzyme has similar effects on adipocyte mitochondria as PLIN5.

**FIGURE 1:**
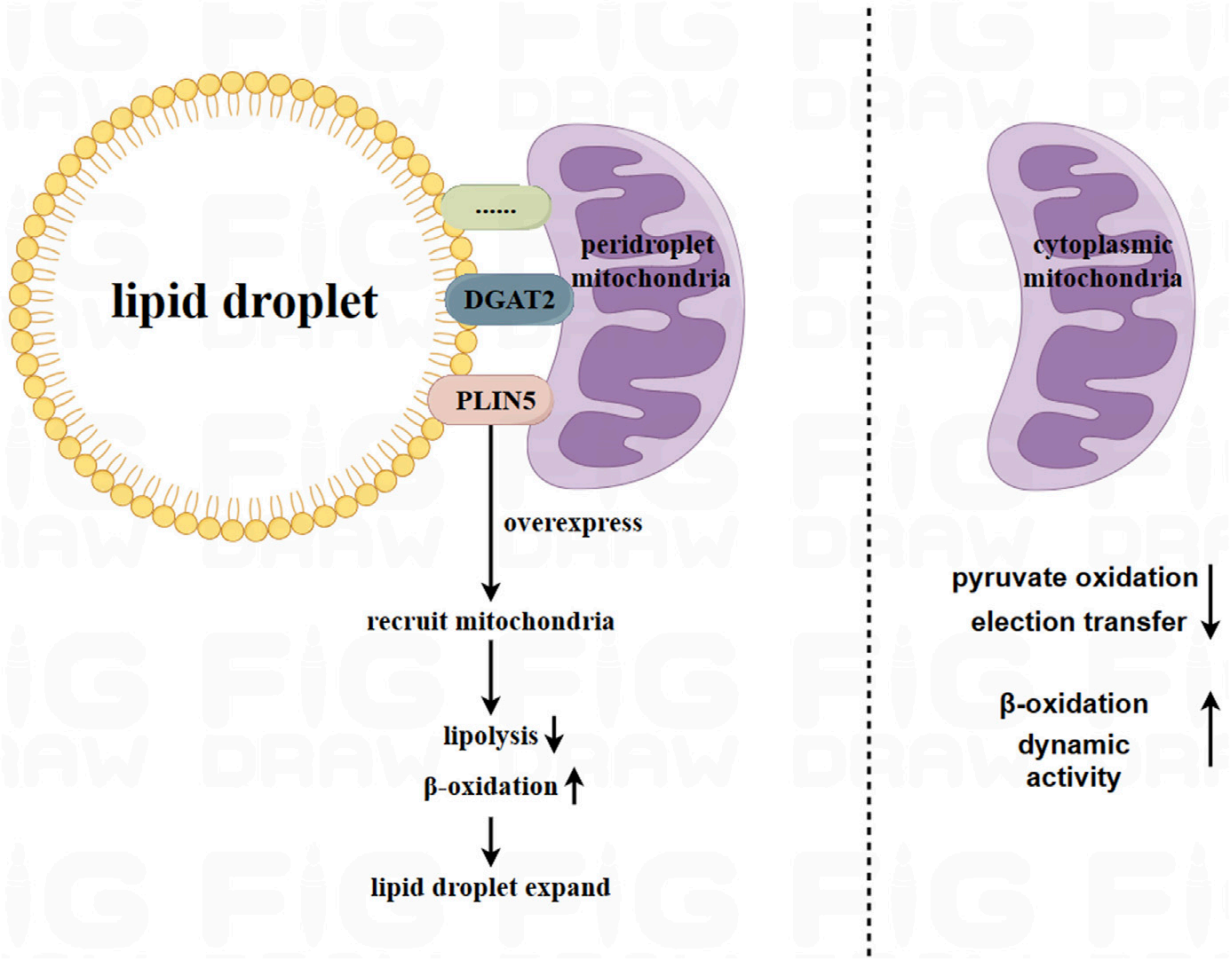
Peridroplet mitochondria (PDM) in white adipocytes and brown adipocytes. Mitochondria are attached to lipid droplets in adipocytes, forming what is known as peridroplet mitochondria (PDM). This attachment is facilitated by proteins such as PLIN5 and DGAT2, as well as other unknown mechanisms. Compared to CM, PDM has a higher capacity for pyruvate oxidation, electron transfer, and ATP synthetic, but lower capacity for β oxidation and dynamic activity. However, PDM may play a key role in expanding lipid droplets.

Peridroplet mitochondria (PDM) and CM differ in their bioenergetics, proteomics, cristae organ, and dynamics (Veliova et al., 2020). Benador et al. separated PDM from brown adipose tissue using high-speed centrifugation. They found that PDM had higher capabilities for pyruvate oxidation, electron transfer, and ATP synthesis compared to CM. However, the β-oxidation and dynamic activity of PDM decreased (Benador et al., 2018). Similarly, Acin Perez et al. found that PDM had higher the ATP synthesis and pyruvate oxidation capabilities compared to CM. On the other hand, CM had higher fatty acid oxidation and uncoupling abilities than PDM (Acin-Perez et al., 2021). Therefore, PDM may play a major role in the expansion of lipid droplets (Benador et al., 2019). Figure 1.

Based on current research, the connection between PDM and lipid droplets is resistant to trypsin digestion and high salt washing. This indicates that the connection between PDM and lipid droplets is not solely attributed to a single protein component (Yu et al., 2015). Further exploration is needed to understand the connection between lipid droplets and PDM.

### 3.2 Adipocyte mitochondria-associated endoplasmic reticulum membrane

Mitochondria-associated endoplasmic reticulum membrane (MAM) is a biochemical and physical connection between the mitochondria and the endoplasmic reticulum. MAM plays a critical role in various physiological processes of adipocytes, including calcium exchange, mitochondrial dynamics, lipid metabolism, mitophagy, and endoplasmic reticulum stress (Fernandes et al., 2021; Wang T. et al., 2022). The formation of MAM is of great significance for adipocytes. Wang et al. suggested that MAM promotes mitochondrial function and maintains the normal redox state, which is essential for preadipocyte differentiation and to maintain mature adipocyte function, including insulin sensitivity and thermogenesis (Wang C. H. et al., 2022).

The connection between mitochondria and the endoplasmic reticulum involves in nearly thousands of proteins and polymer protein complexes, which can be mainly divided into three categories: 1) those specifically located in the MAM; 2) those located in the MAM and other organelles; and 3) those located in the MAM under specific conditions (Wang et al., 2021). Among them, Seipin is a transmembrane protein located in the endoplasmic reticulum and is highly expressed in adipose tissue and the brain (Payne et al., 2008). In mammalian animals, Seipin is located in the MAM and is associated with the calcium regulators IP3R and VDAC in the mitochondria and endoplasmic reticulum through a nutrient-dependent mechanism (Combot et al., 2022). Data showed that mice with a deletion of Seipin suffered from diabetes and hepatic steatosis. They were also unable to tolerate fasting, and exhibited dysfunctional lipid metabolism and disordered thermogenesis ability (Chen et al., 2012; Prieur et al., 2013; Liu L. et al., 2014; Dollet et al., 2016). The calcium levels and respiratory capabilities of adipose tissue in seipin mutant *drosophila* decreased (Ding et al., 2018). In addition to seipin, another protein, PKR-like ER kinase (PERK), has also been reported to be enriched in the MAM (Lebeau et al., 2018). In brown adipocytes, PERK plays a critical role in regulating thermogenesis, maintaining calcium homeostasis, and controlling glucose and lipid metabolism (Kato et al., 2020).

**FIGURE 2:**
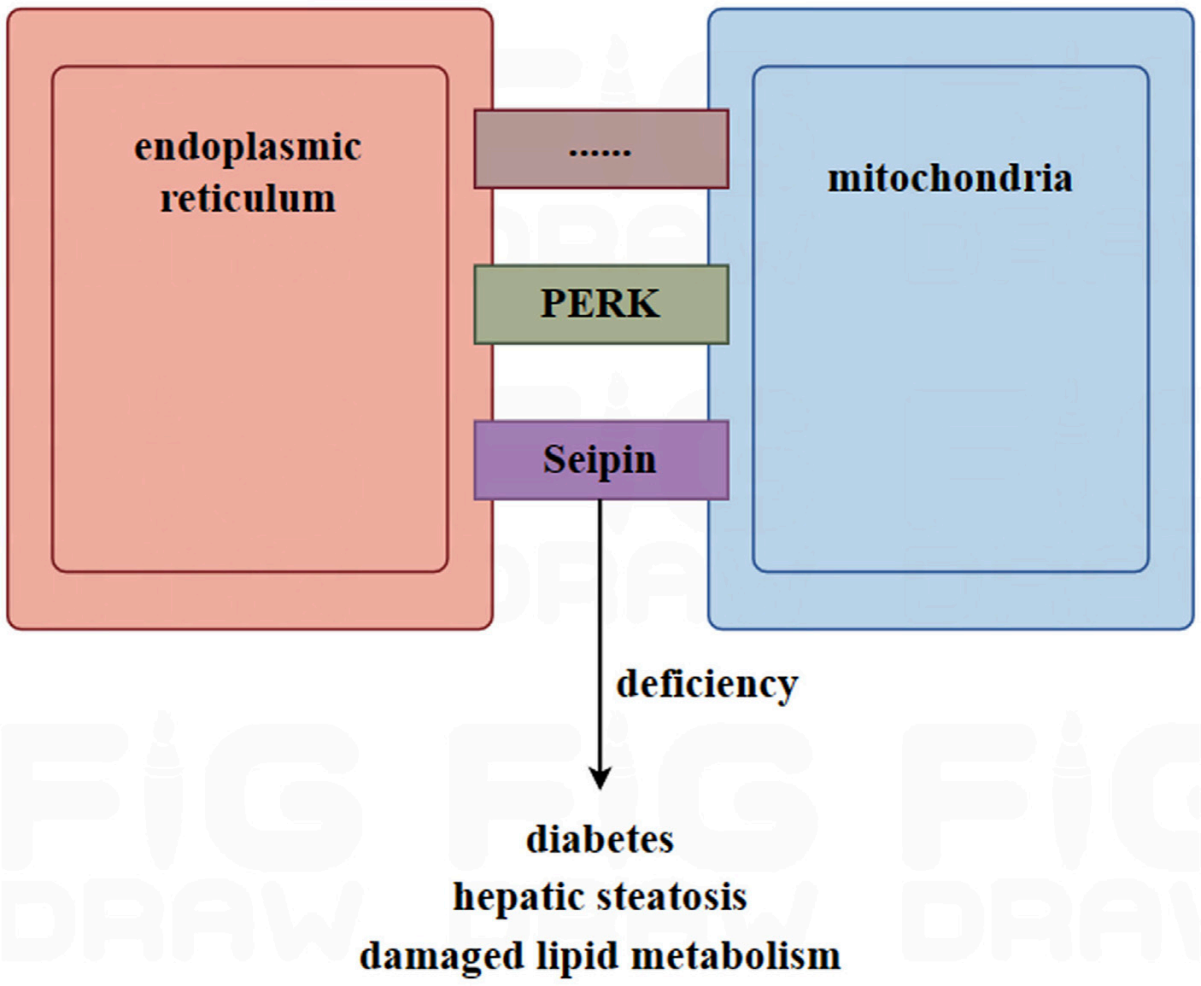
The role of mitochondria-associated endoplasmic reticulum membrane (MAM) in adipocytes. MAM is a site of connection between the mitochondria and the endoplasmic reticulum, facilitated by thousands of proteins, such as Seipin and PERK. In mice, deletion of Seipin induces diabetes, hepatic steatosis, and damaged lipid metabolism. MAM plays a key role in maintaining the homeostasis of calcium in the mitochondria. High-fat diets and obesity can lead to an excessive MAM and overloaded calcium, causing mitochondria to release apoptotic factors into the cytoplasm. This is due to excess the accumulation of calcium in the mitochondria, which ultimately leads to cell apoptosis.

On the other hand, the lumen of the endoplasmic reticulum serves not only as a calcium depository but also as a transport bridge for calcium between the mitochondria and the endoplasmic reticulum. This plays a key role in maintaining the balance of calcium in the mitochondrial (Yang et al., 2020). Figure 2.

According to research, the transport of calcium from the endoplasmic reticulum into mitochondria increased in mice fed a high-fat diet (Feriod et al., 2017). Furthermore, the opening of the mitochondrial permeability transition pore caused by the excessive accumulation of calcium in mitochondria led to the release of apoptotic factors into the cytoplasm (Rizzuto et al., 2012). In mice, obesity was found to induce an increased amount of MAM and calcium overload (Arruda et al., 2014). Furthermore, the mitochondrial enzymes involved in the tricarboxylic acid cycle, which produce ATP, are regulated by calcium. These enzymes include α-ketoglutarate, isocitric acid dehydrogenase, pyruvate dehydrogenase, and ATP synthase (Pallafacchina et al., 2018). Calcium ions serve as second messengers for signal transduction in many cellular processes. Therefore, dysregulated calcium flux is associated with many diseases (Giorgi et al., 2018; McMahon and Jackson, 2018).

## 4 Adipocyte mitochondrial quality control

### 4.1 Adipocyte mitochondria transfer

Numerous studies have shown that extracellular vesicles from several cell types, such as endothelial cells (Tripathi et al., 2019), monocytes (Puhm et al., 2019), adipocytes (Clement et al., 2020; Crewe et al., 2021), and mesenchymal stem cells (Islam et al., 2012; Morrison et al., 2017), contain functional mitochondria or mitochondrial components. Extracellular vesicles that contain functional mitochondria/mitochondrial component can be captured by recipient cells via vesicle-cell fusion (Kitani et al., 2014; Maeda and Fadeel, 2014; Jiang et al., 2016; Torralba et al., 2016; Scozzi et al., 2019).

**FIGURE 3:**
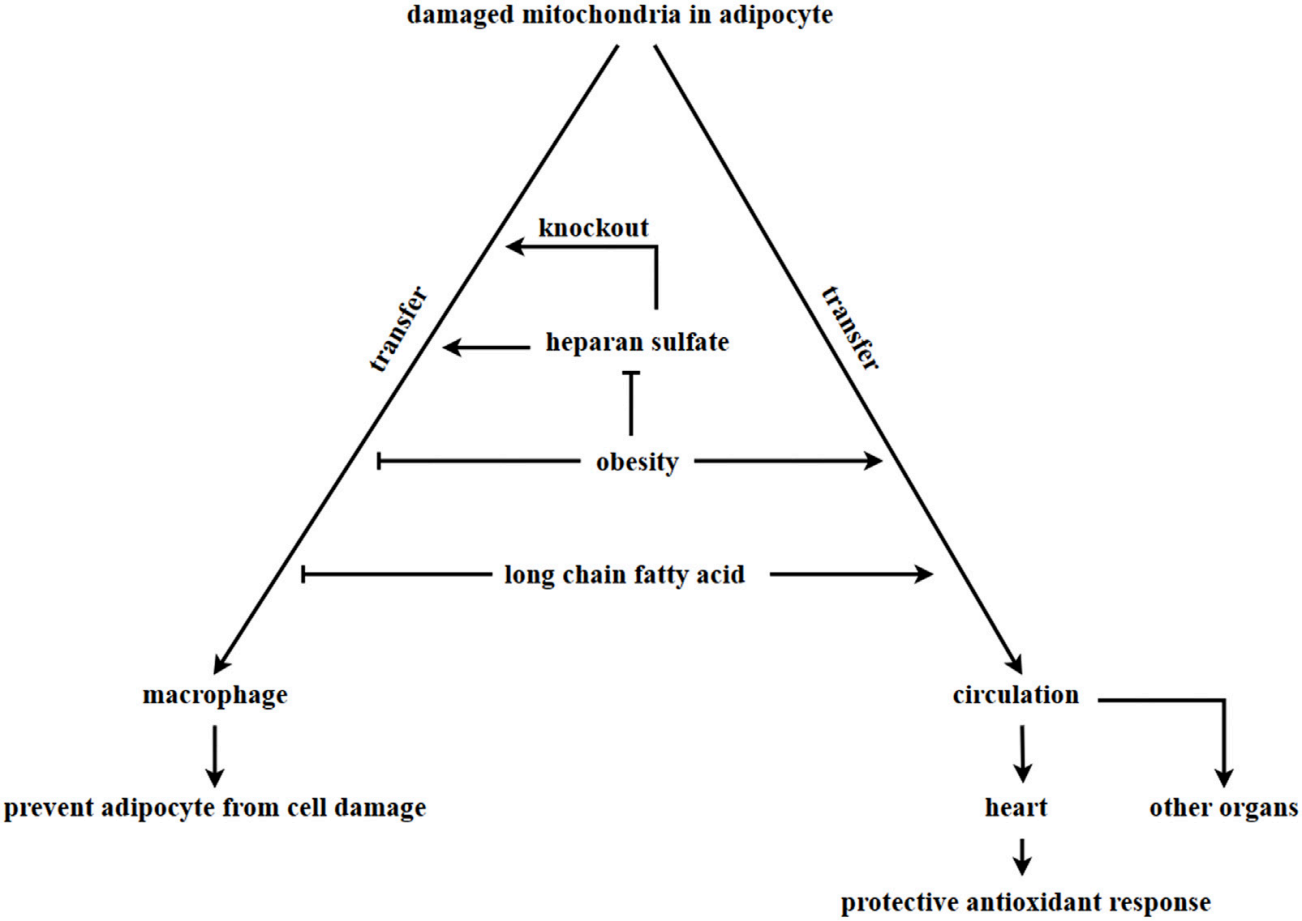
Adipocyte-derived mitochondria transferring. In adipose tissue, damaged mitochondria in adipocytes are transferred to macrophages via vesciles. However, this process is inhibited in obesity, leading to the accumulation of damaged mitochondria in adipocytes. The process of macrophages taking in damaged mitochondria is associated with the HS synthetic pathway. Knock out *Ext1*, which encodes HS, inhibits the ability of macrophages to take in damaged mitochondria. Long-chain fatty acids in a high-fat diet inhibits the transfer of damaged mitochondria into macrophages and promote the transfer of damaged mitochondria into other organs through circulation. It is currently unknown whether adipocytes can uptake in healthy mitochondria to enhance metabolism.

The ρ0 cell line, which is deficient in mitochondrial function, has been shown to uptake in mitochondria from the supernatant, leading to improve cell proliferation (Spees et al., 2006) and restoration of normal mitochondrial respiration (Sinha et al., 2016; Jackson and Krasnodembskaya, 2017; Kim et al., 2018). In adipose tissue, damaged mitochondria from adipocytes are transferred to adipose tissue-resident macrophages. A report (Rosina et al., 2022) suggests that damaged mitochondria resulting from thermogenic stress in brown adipocytes can be transferred to macrophage, which then remove the damaged mitochondria through phagocytosis. Macrophages play a key role in this process. A deficiency in macrophages could lead to an abnormal accumulation of vesicles containing damaged mitochondria in brown adipose tissue, which would impair the thermogenesis of brown adipocytes (Rosina et al., 2022). The synthesis of heparan sulfate (HS) synthetic in macrophages has been linked to the uptake of mitochondria by these cells. A decrease in mitochondria transfer from adipocytes to macrophages in adipose tissue was observed when the expression levels of HS were reduced due to obesity or ablation of the HS synthetic gene *Ext1* (Brestoff et al., 2021). Obese patients are in a state of chronic inflammation, where macrophages are exposed to factors that induce type 1 immune responses, including IFN-γ and LP5. This results in a decreased number of mitochondria transferred from adipocytes to macrophages (Hotamisligil, 2017; Reilly and Saltiel, 2017; Clemente-Postigo et al., 2019). Figure 3.

Additionally, obesity and diet can alter the direction of mitochondrial flow direction derived from adipocytes. The transfer of mitochondria from adipocytes to macrophages was found to be inhibited in cases of obesity. However, mitochondria derived from adipocytes were observed to enter the bloodstream and can be transferred to other organs, such as the heart. This transfer resulted in a heart-protective antioxidant response (Borcherding et al., 2022). According to other research, lard (a long-chain fatty acid) was found to be the main component in a high-lipid diet that inhibits the transfer of mitochondria from adipocytes to macrophages. This component was found to promote the transfer of mitochondria from adipocytes into circulation (Borcherding et al., 2022). In the above discussion, adipocytes always transfer damaged mitochondria out in order to maintain metabolic homeostasis. Several differentiated cells, such as myocardial cells (Acquistapace et al., 2011; Han et al., 2016), endothelial cells (Liu K. et al., 2014), bronchial epithelial cells (Li et al., 2014), corneal epithelial cells (Jiang et al., 2016) and neural cells (Babenko et al., 2015), have been reported to receive mitochondria from mesenchymal stem cells. This transfer of mitochondria helps to prevent apoptosis induced by cell damage. However, it is still unknown whether adipocytes can receive mitochondria from other organs or cells, and the potential effects of extracellular mitochondria entering adipocytes have yet to be explored.

### 4.2 Adipocyte mitochondrial biogenesis

Mitochondrial biogenesis is the process of exciting mitochondrial growth and fission. The markers of mitochondrial biogenesis include the copy number of mtDNA, the ratio of mtDNA to nuclear DNA (nucDNA), and the expression level of mtDNA (Andres et al., 2017; Golpich et al., 2017). It is closely associated with obesity and obesity-related diseases. In cases of obesity, the downregulation of mitochondrial biogenesis, oxidative metabolism pathways, and oxidative phosphorylation proteins occurs in subcutaneous adipose tissue (Heinonen et al., 2015). The expression levels of *PGC-1α*, mtDNA number, and mitochondrial oxidative phosphorylation (OXPHOS) were downregulated in the adipose tissue of obese monozygotic twin pairs (Heinonen et al., 2015). Mitochondrial function and biogenesis are impaired in the subcutaneous adipose tissue of individuals with type 2 diabetes mellitus (Bogacka et al., 2005). Long-term high-fat feeding in mice induced insulin resistance and a significant decrease in mitochondrial biogenesis in visceral adipose tissue (Wang et al., 2014). Mitochondrial biogenesis was found to be facilitated by sports training through an endothelial NO synthase-dependent pathway in both mice and human subcutaneous adipose tissue. This led to an increase in mtDNA content, and insulin-stimulated glucose uptake, and improved lipid metabolism (Trevellin et al., 2014).

**FIGURE 4:**
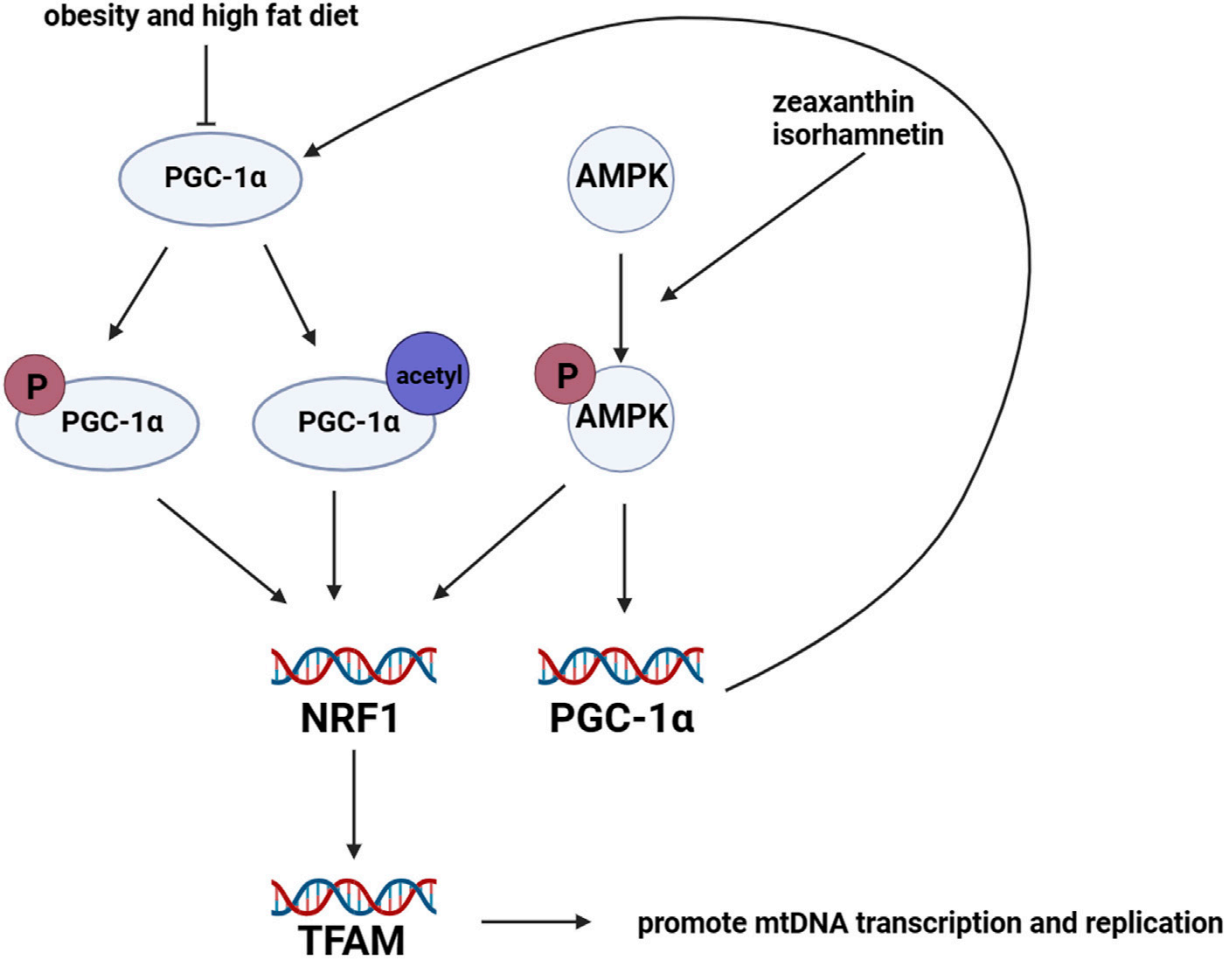
Regulation of mitochondrial biogenesis in adipocytes. Mitochondrial biogenesis in adipocytes is mainly regulated by the lipogenic gene *PGC-1α*. PGC-1α protein is activated through phosphorylation and acetylation. Once activated, PGC-1α promotes the transcription levels of *NRF1* and *NRF2*, which in turn leads to the upregulation of *TFAM*. TFAM promotes the transcription and replication of mtDNA, resulting in mitochondrial biogenesis. However, the expression of PGC-1α is downregulated in mice with obesity and those fed a high-fat diet. Furthermore, the activation of AMPK promotes the transcription levels of *NRFs* and *PGC-1α*.

Mitochondrial biogenesis is regulated by adipogenic genes (Rosen and Spiegelman, 2000), including peroxisome proliferator receptor γ coactivator 1α (PGC-1α) (Spiegelman et al., 2000), peroxisome proliferator-activated receptor γ (PPARγ) (Spiegelman et al., 2000; Rosen and Spiegelman, 2001), CCAAT/enhancer-binding protein α (C/EBP) (Rosen and Spiegelman, 2001), cAMP-response element binding protein (CREB) (Vankoningsloo et al., 2006), and estrogen-related receptor α (ERRα) (Ijichi et al., 2007). Among these regulators, PGC-1α is considered the primary regulator of mitochondrial biogenesis (Puigserver and Spiegelman, 2003). PGC-1α can be activated through phosphorylation or acetylation, which induces the expression of nuclear respiratory factor 1 and 2 (NRF1 and NRF2). This, in turn, promotes the expression of mitochondrial transcription factor A (TFAM) (Scarpulla, 2002; Scarpulla, 2011). TFAM is responsible for inducing transcription and replication of mtDNA, which facilitates mitochondrial biogenesis (Wood Dos Santos et al., 2018). Furthermore, an increasing amount of research has shown that AMP-activated protein kinase (AMPK) plays a critical role in mitochondrial biogenesis. Mitochondrial biogenesis is promoted by the protein expression of PGC-1α and NRFs, which are induced by activated AMPK (Canto and Auwerx, 2009). In pharmacological experiments, isorhamnetin (3-O-methylquercetin) was found to promote mitochondrial biogenesis by activating AMPK in 3T3-L1 preadipocytes (Lee and Kim, 2018). Additionally, the administration of zeaxanthin, which is an oxygenated carotenoid, significantly increased mtDNA content and mRNA expression levels of genes related to mitochondrial biogenesis by activating AMPK in 3T3-L1 preadipocytes. This activation promoted mitochondrial biogenesis and the browning of adipocytes (Liu et al., 2019). Figure 4.

On the other hand, a different report has identified a significant positive correlation between mtDNA copy number and the rate of fat production in human adipocytes (Kaaman et al., 2007). Furthermore, the upregulation and downregulation of mitochondrial biogenesis directly enforce or inhibit the synthesis and secretion of adiponectin in adipocytes (Koh et al., 2007). Another research has reported that promoting mitochondrial biogenesis can enhance oxidative phosphorylation capacity, reduce pathological oxidative stress, and repair mitochondrial dysfunction (Cameron et al., 2017). Therefore, exploring the relationship between mitochondrial biogenesis and adipocyte physiology is of great significance.

### 4.3 Adipocyte mitochondrial autophagy

Autophagy is a cellular process in eukaryotic cells that involves the removal of damaged components and is regulated by autophagy-related genes. Autophagy can be classified based on its selectivity into different types, including lipid droplet autophagy (lipophagy), mitochondrial autophagy, peroxisome autophagy (pexophagy), ribosome autophagy (ribophagy), and endoplasmic reticulum autophagy (reticulophagy) (Gatica et al., 2018). Mitophagy is the process of removing damaged mitochondria, which is an important mechanism for maintaining mitochondrial quality (Youle and Narendra, 2011; Bratic and Larsson, 2013). Aging-related diseases, including obesity, can be induced if dysfunctional mitochondria are not cleared by mitophagy (Green et al., 2011).

Mitophagy can be subdivided into two types: ubiquitin-mediated mitophagy and receptor-mediated mitophagy (Li et al., 2022). Ubiquitin-mediated mitophagy involves the PTEN-induced putative kinase 1 (PINK1)/Parkin-mediated pathway, as well as other ubiquitin-mediated pathways (Li et al., 2022). Research has reported that the transcription level of *PINK1* is negatively correlated with the risk of diabetes in obesity (Franks et al., 2008). In mice, depletion of PINK1, the core regulator of mitophagy, in either the whole body or brown adipose tissue, resulted in brown adipose tissue dysfunction and a tendency towards obesity (Ko et al., 2021). However, mild decreases in mitophagy in adipose tissue-specific knockout of *PARK2* mice increased mtDNA content and improved mitochondrial function. This promoted mitochondrial biogenesis by increasing PGC-1α protein stability and protected mice against diet-induced or aging-induced obesity (Moore et al., 2022).

Receptor-mediated mitophagy involves several pathways, including the BCL2/adenovirus BCL2-interacting protein 2 (BNIP2)-mediated pathway, the FUN14 domain containing 1 (FUNDC1)-mediated pathway, and the lipid-mediated pathway (Li et al., 2022). Serine/threonine protein kinases 3 and 4 (STK3 and STK4), whose expression levels are upregulated in obesity, can promote mitophagy by regulating the phosphorylation and dimerization state of BNIP3. The metabolism characteristics of obese mice were improved by pharmacological inhibition of STK3 and STK4 (Cho et al., 2021). Furthermore, the deletion of FUNDC1, a mitophagy receptor, can lead to a mitophagy disorder, worsening adipose tissue inflammation and exacerbating diet-induced obesity (Wu et al., 2019). The PGC-1α/NRF1 pathway regulates FUNDC1-mediated mitophagy. NRF1 facilitates the expression of FUNDC1 by binding to the promoter of the *FUNDC1* gene. Knockout of *FUNDC1* leads to the accumulation of damaged mitochondria, which impairs adaptive thermogenesis in brown adipose tissue (Liu et al., 2021).

### 4.4 Adipocyte mitochondrial dynamics

In a physiological environment, mitochondria maintain homeostasis through the processes of fission and fusion. Damaged and healthy mitochondria undergo changes in their components through mitochondrial fission, while damaged mitochondria discharge dysfunctional components through mitochondrial fusion (Twig et al., 2008).

Outer mitochondrial membrane (OMM) fusion is regulated by Mitofusin 1 and 2 (MFN1 and MFN2), which are located in the OMM (Santel and Fuller, 2001). The connection between mitochondria depends on MFN1, which contains the HR domain (Galloway and Yoon, 2013). On the other hand, MFN2 interacts with itself and recruits MFN1, leading to heterooligomerization that promotes mitochondrial fusion (Detmer and Chan, 2007). Moreover, MFN2 regulates the interaction between mitochondria and lipid droplets or the endoplasmic reticulum, thereby regulating energy metabolism and calcium signaling in adipocytes (de Brito and Scorrano, 2008; Boutant et al., 2017; Mancini et al., 2019). Furthermore, PGC-1α collaboratively activates estrogen receptor-related receptors to regulate both MFN1 and MFN2 (Elezaby et al., 2015). However, dysfunction of MFN2 inhibits oxidative phosphorylation complexes I, II, III, and V, thereby inhibiting the oxidation of pyruvate, glucose, and fatty acids, and decreasing mitochondrial membrane potential (Pich et al., 2005). In humans, a mutation in *MFN2* inhibits the expression of leptin and induces mitochondrial dysfunction in adipocytes (Rocha et al., 2017).

The regulation of IMM fusion is primarily controlled by OPA1, a protein that plays a crucial role in preserving the structure of mitochondrial cristae (Otera and Mihara, 2011). Severe changes in the mitochondrial network, such as mitochondrial fragmentation, dispersion, and fracturing and disorder of mitochondrial cristae, are induced by the dysregulated expression of *OPA1* (Griparic et al., 2004). However, OPA1 can be inactivated by the zinc ion metalloproteinase (OMA1), a protein located in the inner mitochondrial membrane. When OPA1 is inactivated, it further inhibits mitochondrial fusion in mammalian cells under stress (Head et al., 2009). Moreover, the deletion of *OMA1* leads to weight gain and fatty liver degeneration. An abnormal OMA1-OPA1 system affects the thermogenesis and metabolism of brown adipose tissue, suggesting a link between an abnormal OMA1-OPA1 system and obesity as well as related diseases (Quiros et al., 2013).

Mitochondrial fission is mainly regulated by two proteins: dynamin-related protein 1 (DRP1) and fission protein 1 (FIS1) (Rovira-Llopis et al., 2017). Circular DRP1 constricts the mitochondrial membrane through a GTP-dependent pathway to facilitate mitochondrial fission, while FIS1 regulates mitochondrial fission by recruiting DRP1 (Rovira-Llopis et al., 2017). In addition to FIS1, mitochondrial fission factor (MFF) and mitochondrial dynamics proteins of 49 kDa (MiD49) and MiD51, which are located in the OMM, are also involved in recruiting DRP1 (Loson et al., 2013).

**FIGURE 5:**
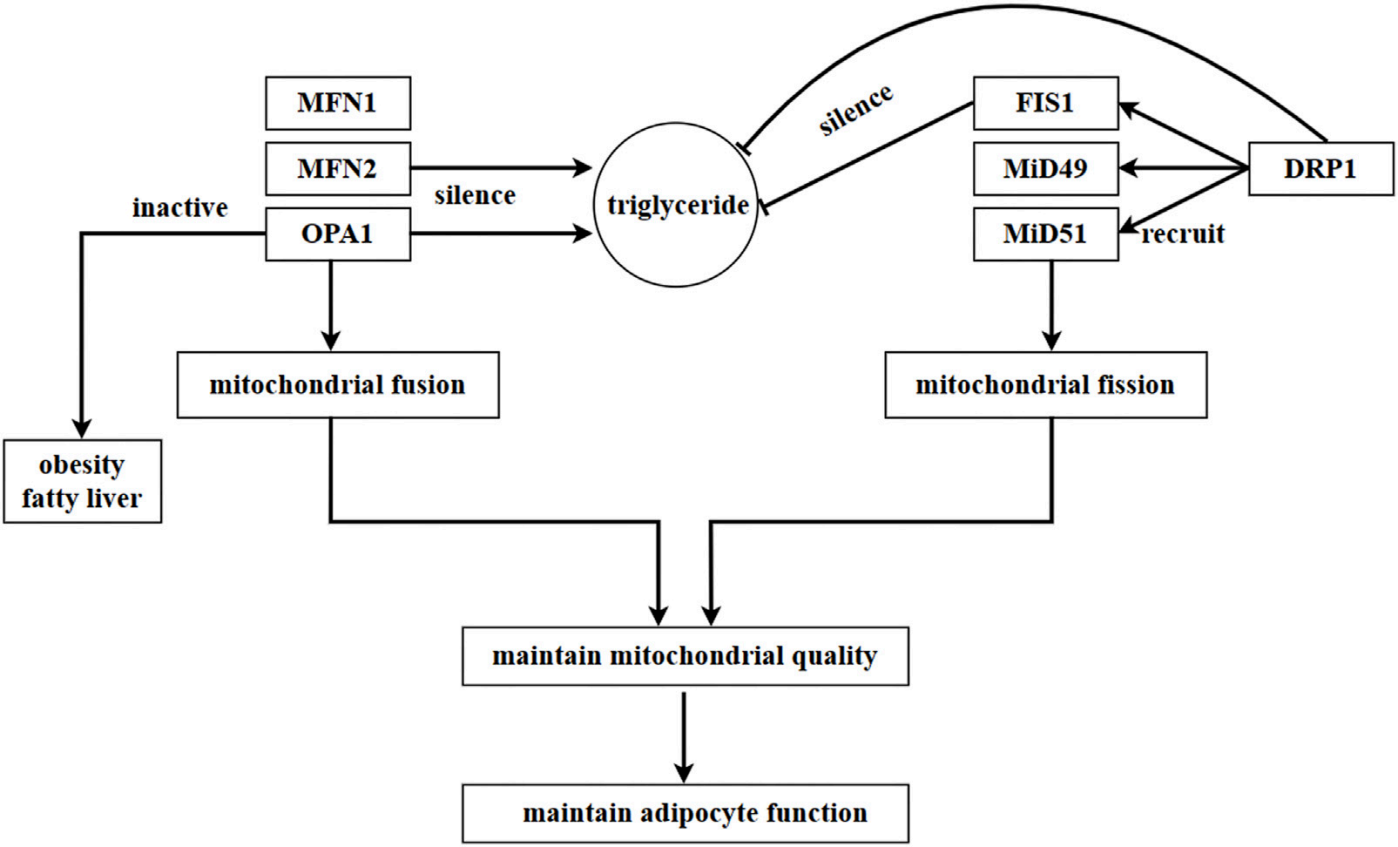
Regulation of mitochondrial dynamics in adipocytes. In adipocytes, mitochondrial fusion is mainly regulated by MFN1, MFN2, and OPA1, while mitochondrial fission is mainly regulated by DRP1. DRP1 is recruited by proteins located in OMM, such as FIS1, MiD49, and MiD51. In adipocytes, a mutation in *MFN2* inhibits the expression of *leptin*. Triglyceride content increases as a result of the silence of *MFN2* and *OPA1*, while it decreases due to the silence of FIS1 and DRP1. This suggests that the accumulation of triglycerides is regulated by mitochondrial dynamics in adipocytes. Moreover, SIRT3 and DRP1 promote the browning of white adipocytes, and this process is impeded by the inhibition or knockdown of SIRT3 or DRP1.

The data shows that silencing FIS1 and DRP1 resulted in a decrease in triglyceride levels, whereas silencing MFN2 and OPA1 in adipocytes induced an increase in triglyceride levels. This suggests that mitochondrial dynamics play a role in regulating the accumulation of triglycerides (Kita et al., 2009). A pharmacological experiment demonstrated that ellagic acid facilitated the browning of cold-exposed white adipocytes by increasing mitochondrial dynamics-related factors, such as SIRT3, NRF1, CPT1β, DRP1, and FIS1. However, the effect of ellagic acid was blocked by the DRP1 inhibitor Mdivi-1 or knockdown of SIRT3 (Park et al., 2021). These findings further confirm the close association between adipocyte physiology and mitochondrial dynamics. On the other hand, mitochondrial bioenergetics are improved, and insulin sensitivity is facilitated through mitochondrial fission and fusion in adipose tissue (Tol et al., 2016). Figure 5.

Mitochondrial function is dependent on the quality control of mitochondria, in which mitochondrial dynamics play a critical role. Adipocytes are cells with a high level of energy metabolism, and mitochondria serve as the primary sites for energy metabolism within adipocyte. Therefore, maintaining mitochondrial quality control through dynamic fusion and fission is essential for proper adipocyte function.

## 5 The changes of adipose mitochondrial metabolism in obesity-related metabolic diseases

Adipose tissue mitochondrial metabolism is damaged in obesity. Research has shown that in morbidly obese women, adipogenesis and fatty acid oxidation are downregulated in subcutaneous adipose tissue (SAT), while both remain unchanged in visceral adipose tissue (VAT). This suggests that SAT may decrease the expression of genes related to adipogenesis and fatty acid oxidation to limit further adipose generation, while VAT may not have this ability (Auguet et al., 2014). The protein levels of adipose triglyceride lipase (ATGL) and hormone sensitive lipase (HSL) were found to be decreased in the adipose tissue of individuals with obesity and insulin resistance (Jocken et al., 2007). Accordingly, fat mass and lipid accumulation increased in insulin-sensitive tissues in whole-body knockout mice for ATGL and HSL (Girousse and Langin, 2012). Lipid accumulation was inhibited in human multipotent adipose-derived stem cells through genetic and pharmacological knockdown of ATGL and/or HSL. This resulted in the induction of insulin resistance and decreased mitochondrial oxygen consumption, as well as damage to the PPARγ signal (Jocken et al., 2016). Compared to selectively bred obesity-resistant rats, the mRNA level of *CPT1b* was lower in SAT of obesity-prone rat. This may be the reason for fat accumulation (Ratner et al., 2015). Accordingly, overexpression of *CPT1a* induced enforced fatty acid oxidation, improved insulin sensitivity, and decreased inflammation in 3T3-L1 preadipocytes (Gao et al., 2011). In individuals with obesity and metabolic disease, mitochondrial dysfunction in brown adipose tissue leads to a decrease in fatty acid oxidation and energy consumption. This dysfunction also induces ectopic accumulation of fat (de Mello et al., 2018). In patients with insulin resistance, lipolysis is increased in adipose tissue while lipogenesis is damaged. This leads to the release of cytokines and lipid metabolites, which further exacerbate insulin resistance (Bodis and Roden, 2018). On the other hand, insulin resistance is associated with an increase in lipid accumulation in the liver and muscle, as well as a decrease in the lipid storage ability of adipose tissue (Toledo and Goodpaster, 2013). Therefore, obesity is one of the main causes of insulin resistance.

Proteomics research has shown a negative correlation between BMI and four important mitochondrial proteins (citrate synthase, HADHA, LETM1, and mitofilin) in human omental adipose tissue (Lindinger et al., 2015). Similarly, compared to non-obese individuals, individuals with obesity showed a significant decrease in oxygen consumption and citrate synthase activity in adipocytes and adipose tissue from omental and SAT of obesity. However, there was no significant change in mitochondrial amount between the two groups (Yin et al., 2014). The main genes involved in OXPHOS, TCA cycle and fatty acid oxidation were found to be downregulated in both white adipose tissue (WAT) and brown adipose tissue (BAT) of mice with diabetes and those fed a high-fat diet. However, the expression of these genes was restored in a dose-dependent manner by rosiglitazone (Rong et al., 2007). In mice fed a high-fat diet, depletion of ATP through knockout of fumarate hydratase in white and brown adipose tissue resulted in decreased fat mass and smaller adipocytes. This protected the mice against obesity, insulin resistance, and fatty liver (Yang et al., 2016). Furthermore, compared to mice fed a low-fat diet, those fed a high-fat diet showed a significant increase in whole-body fat, as well as exacerbated glucose and insulin intolerance (Cummins et al., 2014).

Adipose tissue’s mitochondrial energy metabolism is impaired in obesity related metabolic diseases. The data shows that obesity is associated with a decreased level of oxidative phosphorylation complex I and IV, as well as decreased mitochondrial oxygen consumption (Fischer et al., 2015). In cases of obesity, the activity of complex I and IV, and the mitochondrial transmembrane potential, are decreased (Chattopadhyay et al., 2011). In mouse models of diet-induced and genetically regulated obesity, the oxidative phosphorylation capacity of white adipocytes was found to be limited (Schottl et al., 2015). Furthermore, the activity of complex IV in white adipocytes from mice and VAT from humans was found to decrease with aging (Soro-Arnaiz et al., 2016). Compared to their lean co-twins, individuals with obesity showed downregulation in the gene expression levels of the mitochondrial oxidative pathway, and the protein levels of OXPHOS in their SAT (Heinonen et al., 2015). In patients with non-alcoholic fatty liver (NAFL) and non-alcoholic steatohepatitis (NASH), a significant decrease in mitochondrial maximal respiratory capacity was observed, along with decreased insulin sensitivity. Additionally, the expression level of complex IV was decreased in SAT from patients with NASH (Pafili et al., 2022). On the other hand, adipocyte function is influenced by a damaged mitochondrial OXPHOS pathway. Inhibition of complex III resulted in mitochondrial dysfunction, leading to abnormal triglyceride accumulation, decreased expression of adipogenic markers, and damaged differentiation of 3T3-L1 preadipocytes (Vankoningsloo et al., 2006). Consistently, the inhibition of complex I induced damaged differentiation of cells, decreased ATP synthesis, and downregulated expression of adipogenic genes such as *LPL*, *PPARγ*, *C/EBPα*, and *SREBP-1c*. (Lu et al., 2010). The growth of 3T3-L1 preadipocytes was inhibited by inhibitors of complex I and ATP synthase (Carriere et al., 2003).

Research has shown that proinflammatory cytokines play different roles in regulating adipocytes metabolism. For example, TNFα enhances mitochondrial basic respiration, while IL-6 and IL-1β decrease the mitochondrial maximal respiratory capacity (Hahn et al., 2014). In cases of obesity, various cytokines activate the macrophages in white adipose tissue, leading to chronic inflammation. This inflammation can cause dysregulation of lipid, glucose, and energy metabolism in adipocytes (Hotamisligil, 2017; McLaughlin et al., 2017; Reilly and Saltiel, 2017; Larabee et al., 2020). In conclusion, the mitochondrial metabolism of adipocyte is impaired in obesity-related metabolic diseases. On the other hand, damaged mitochondrial metabolism can affect the normal function of adipose tissue, and ultimately impacting the overall metabolic health of the body (Heinonen et al., 2020). Table 1.

**TABLE 1:** The changes of adipose mitochondrial metabolism in obesity-related metabolic diseases.

Species	Disease	Tissue/cell	The changes of mitochondrial metabolism	References
Human	Obesity	Omental and SAT	Reduced oxygen consumption and citrate synthase	Yin et al. (2014)
Human	Obesity	Adipose tissue	Fewer complex I and IV components	Fischer et al. (2015)
Human	Obesity	SWAT	Reduced mitochondrial transmembrane potential and respiratory chain complex activity	Chattopadhyay et al. (2011)
Human	Obesity	SAT	Downregulated expression level and protein level of OXPHOS pathway	Heinonen et al. (2015)
Human	Morbid obesity	SAT	Downregulated adipogenesis and fatty acid oxidation	Auguet et al. (2014)
Human	Obesity and insulin resistance	AT	Decreased level of ATGL and HSL	Jocken et al. (2007)
Human	Insulin resistance	WAT	Enforced lipolysis and impaired adipogenesis	Bodis and Roden (2018)
Human	Non-alcoholic fatty liver and steatohepatitis	SAT	Reduced mitochondrial maximal respiratory capability and insulin sensitivity and expression level of complex IV	Pafili et al. (2022)
Mice	High-fat diet fed	WAT	Increased whole-body fat mass, reduced respiratory exchange ratio and glucose and insulin sensitivity	Cummins et al. (2014)
Mice	Obese-prone	WAT	Decreased expression of *CPT1b*	Ratner et al. (2015)
Mice	Obesity	White adipocytes	Reduced mitochondrial respiratory capability and limited OXPHOS capability	Schottl et al. (2015)
Mice	Diabetes and high-fat diet fed	SAT (BAT and WAT)	Suppressed expression of majority of the OXPHOS/TCA/FAO genes	Rong et al. (2007)

## 6 Conclusion

Mitochondria are crucial organelles within cells that produce ATP through oxidative phosphorylation pathways to maintain adipocyte metabolism. Mitochondria are also vital organelles for maintaining the redox state of adipocytes. Excessive accumulation of ROS leads to the release of apoptotic factors into the cytoplasm, which in turn induces apoptosis of adipocytes. Besides, mitochondria serve as storage sites for calcium ions. Calcium plays a crucial role as a secondary signal in many physiological processes. Mitochondria are responsible for maintaining calcium homeostasis by facilitating the intake of calcium through membrane synergistic transporters and releasing calcium through sodium-calcium exchange systems and membrane transport channels. Interestingly, mitochondria are associated with other organelles, such as lipid droplets and the endoplasmic reticulum, in adipocytes. This association improves mitochondrial function and is beneficial for the development and function of adipocytes. Mitochondrial quality control, achieved through mitochondrial dynamic fission and fusion, biogenesis, mitophagy, and transfer, is vital for adipocytes to respond to changes in an organism’s environment. In conclusion, it is of great significance to understand the relationship between mitochondrial physiology and adipocytes/adipose tissue.

## Abbreviations

AMPK, AMP-activated protein kinase; ATGL, Adipose triglyceride lipase; ATPase, ATP synthase; BNIP2, BCL2/adenovirus BCL2-interacting protein 2; BAT, Brown adipose tissue; CM, Cytoplasmic mitochondria; C/EBP, CCAAT/enhancer-binding protein α; DRP1, Dynamin-related protein 1; ERRα, Estrogen-related receptor; FUNDC1, FUN14 domain containing 1; FIS1, Fission protein 1; HS, Heparan sulfate; IMM, Inner mitochondrial membrane; MAM, Mitochondria-associated endoplasmic reticulum membrane; MCL1, Myeloid cell leukemia 1; MFF, mitochondrial fission factor; MFN, Mitofusin; MiD49, Mitochondrial dynamics proteins of 49kda; mitophagy, Mitochondria autophagy; mtDNA, Mitochondrial DNA; NAFA, Non-alcohol fatty liver; NASH, Non-alcohol steatohepatitis; NEIL1 and NEIL2, Nei like DNA glycosylase 1 and 2; nucDNA, Nuclear DNA; NRF1, Nuclear respiratory factor 1; NTH1, *N*th like DNA glycosylase 1; OGG1, Oxoguanine DNA glycosylase 1; OMA1, Zinc ion metalloproteinase; OMM, Outer mitochondrial membrane; OPA1, Optic atrophy 1; OXPHOS, Oxidative phosphorylation; PDM, Peridroplet mitochondria; pexophagy, Peroxisome autophagy; PGC-1α, Peroxisome proliferator receptor γ coactivator 1α; PHB1, Prohibitin 1; PINK1, PTEN-induced putative kinase 1; PLIN5, perilipin 5; PPARγ, Peroxisome proliferator-activated receptor γ; reticulophagy, Endoplasmic reticulum autophagy; SAT, Subcutaneous adipose tissue; ribophagy, Ribosome autophagy; SLP2, stomatin-like protein 2; STK, Serine/threonine protein kinase; TFAM, Mitochondrial transcription factor A; UCP1, Uncoupling protein 1; VAT, Visceral adipose tissue; WAT, White adipose tissue.
